# Targeting RING domains of Mdm2–MdmX E3 complex activates apoptotic arm of the p53 pathway in leukemia/lymphoma cells

**DOI:** 10.1038/cddis.2015.358

**Published:** 2015-12-31

**Authors:** W Wu, C Xu, X Ling, C Fan, B P Buckley, M V Chernov, L Ellis, F Li, I G Muñoz, X Wang

**Affiliations:** 1 Department of Pharmacology and Therapeutics, Roswell Park Cancer Institute, Buffalo, NY, USA; 2 Department of Stress Biology, Small Molecule Screening Core Facility, Roswell Park Cancer Institute, Buffalo, NY, USA; 3 Crystallography Unit, Structural Biology and Biocomputing Programme, Spanish National Cancer Research Centre (CNIO), Melchor Fernández Almagro 3, Madrid, Spain

## Abstract

Reactivation of tumor-suppressor p53 for targeted cancer therapy is an attractive strategy for cancers bearing wild-type (WT) *p53*. Targeting the Mdm2–p53 interface or MdmX ((MDM4), mouse double minute 4)–p53 interface or both has been a focus in the field. However, targeting the E3 ligase activity of Mdm2–MdmX really interesting new gene (RING)–RING interaction as a novel anticancer strategy has never been explored. In this report, we describe the identification and characterization of small molecule inhibitors targeting Mdm2–MdmX RING–RING interaction as a new class of E3 ligase inhibitors. With a fluorescence resonance energy transfer-based E3 activity assay in high-throughput screening of a chemical library, we identified inhibitors (designated as MMRis (Mdm2–MdmX RING domain inhibitors)) that specifically inhibit Mdm2–MdmX E3 ligase activity toward Mdm2 and p53 substrates. MMRi6 and its analog MMRi64 are capable of disrupting Mdm2–MdmX interactions *in vitro* and activating p53 in cells. In leukemia cells, MMRi64 potently induces downregulation of Mdm2 and MdmX. In contrast to Nutlin3a, MMRi64 only induces the expression of pro-apoptotic gene PUMA (p53 upregulated modulator of apoptosis) with minimal induction of growth-arresting gene p21. Consequently, MMRi64 selectively induces the apoptotic arm of the p53 pathway in leukemia/lymphoma cells. Owing to the distinct mechanisms of action of MMRi64 and Nutlin3a, their combination synergistically induces p53 and apoptosis. Taken together, this study reveals that Mdm2–MdmX has a critical role in apoptotic response of the p53 pathway and MMRi64 may serve as a new pharmacological tool for p53 studies and a platform for cancer drug development.

Activation of tumor-suppressor p53 as a targeted non-genotoxic cancer therapy has been pursued for many years,^1, 2^ because p53 possesses potent tumor-suppressing activity *in vivo*.^3, 4, 5^ p53 can inhibit cancer cell growth by cell cycle arrest or terminate their proliferation by inducing apoptosis and senescence.^6^ The p53-based therapy is particularly attractive for cancer types including retinoblastoma, neuroblastoma and leukemia/lymphoma in which p53 is rarely mutated^7^ and p53-dependent apoptotic pathway is a predominant endpoint.^8, 9, 10^ Except for cancer-selected p53 mutations, the p53 activity is mainly inhibited by p53-binding proteins Mdm2 and MdmX ((MDM4), mouse double minute 4) in normal and cancer cells.^11, 12^ Prior focus of p53 reactivation strategy has been on targeting the Mdm2–p53 and/or MdmX–p53 interface. This has led to the discovery of a list of potent Mdm2–p53 inhibitors^13^ with several compounds of this class being advanced to phase I clinical trials in hematological neoplasia and solid tumors.^2^ However, the therapeutic effects of these Mdm2–p53 inhibitors can be attenuated by MdmX overexpression.^14, 15, 16^ Although peptide inhibitors with dual functions of inhibiting both Mdm2–p53 and MdmX–p53 interactions will overcome this problem and enhance p53-dependent cancer killing;^17, 18^ these inhibitors will not inhibit Mdm2 E3 ligase activity toward non-p53 targets such as retinoblastoma protein (RB), p21 and DAXX (death domain-associated protein),^19, 20, 21^ which to a different extent contributes to the p53-dependent biological effects.

Recent genetic studies indicated that really interesting new gene (RING) domains of Mdm2 and MdmX are required for *in vivo* inhibition of p53 activity during development.^22, 23, 24^ MdmX was reported to stimulate Mdm2-mediated p53 multiple monoubiquitination using glutathione *S*-transferase (GST) fusion Hdm2 proteins.^25, 26^ Using non-GST Hdm2 proteins in *in vitro* biochemical assays, we found that MdmX–Mdm2 RING–RING interaction is essential for p53 polyubiquitination and proteasome-dependent degradation.^26^ These findings established that Mdm2–MdmX complex is the key regulator of p53 activity and Mdm2–MdmX RING–RING interaction is a critical but an unexplored interface for drug targeting.^27^ Identification of E3 ligase inhibitors for cancer therapy presents a huge opportunity but with great challenges.^28^ In this report, we describe successful identification and characterization of small molecule inhibitors for the E3 ligase activity of Mdm2–MdmX E3 complex. Among seven specific MMRis (Mdm2–MdmX RING domain inhibitors), MMRi64 was followed up in detail in this report. MMRi64 has several unique features that distinguish it from Mdm2–p53 inhibitor Nutlin3a. MMRi64 disrupts Mdm2–MdmX interaction *in vitro* and inhibits the E3 ligase activity of Mdm2–MdmX without affecting the E3 ligase activity of Mdm2 RING domain homodimers. MMRi64 induces p53 accumulation without induction of Mdm2 and p21 in lymphoma cells, which is distinct from the effects of Nutlin3a. Finally, MMRi64 induces PUMA (p53 upregulated modulator of apoptosis) but strongly downregulates MdmX and Mdm2, consequently activating the apoptotic arm of the p53 pathway in leukemia/lymphoma cells without the induction of growth arrest.

## Results

### High-throughput screening of small molecule inhibitors for the E3 ligase activity of Mdm2–MdmX E3 complex

**Figure 1:**
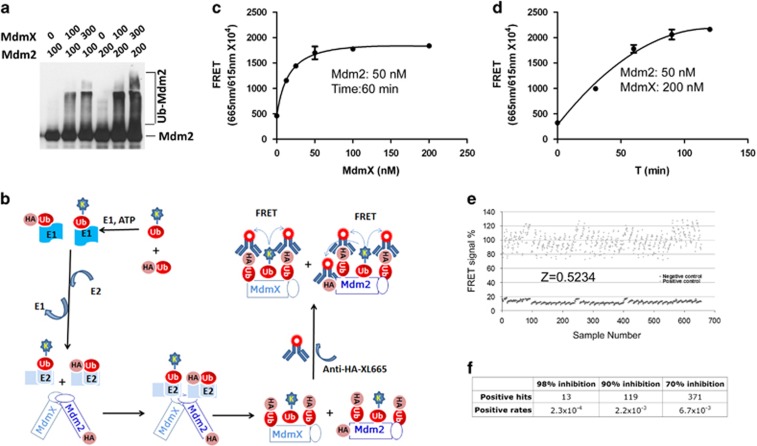
HTS of small molecule inhibitors of Mdm2–MdmX E3 ligase activity. (**a**) Concentration-dependent effect of Mdm2 and MdmX on Mdm2 ubiquitination. *In vitro* ubiquitination reaction performed with indicated concentrations (nM) of Mdm2 and MdmX recombinant proteins followed by WB for Mdm2. Ubiquitinated Mdm2 (Ub-Mdm2) and Mdm2 bands were shown. (**b**) Schematic illustration of FRET-based assay of Mdm2 and MdmX ubiquitination. Two fluorophores that generate FRET were conjugated to ubiquitin (Ub-K, ubiquitin cryptate) and anti-HA antibody (anti-HA-XL665). These two fluorophores will be brought in proximity for FRET to occur once ubiquitin chains are assembled on HA-Mdm2 and MdmX proteins. (**c**) MdmX concentration-dependent stimulation of FRET signals under fixed concentration of Mdm2 and reaction time. (**d**) Reaction time-dependent increase of FRET signals at fixed concentrations of Mdm2 and MdmX proteins. (**e**) Z-score calculation with FRET data from 650 samples. (**f**) Summary of positive hits at different cutoffs of inhibition of FRET signals by compounds after completion of HTS of Chembridge DIVERSet library

We previously reported that Mdm2–MdmX RING–RING interaction is required for p53 polyubiquitination.^26^ This interaction also stimulates Mdm2 autoubiquitination and MdmX ubiquitination (Figure 1a and Wang *et al.*^26^). To establish a biochemical assay for screening small molecule inhibitors of Mdm2–MdmX RING–RING interaction, we took advantage of an *in vitro* assay for MdmX-stimulated Mdm2 autoubiquitination as a readout of the interaction effect. To facilitate its application in high-throughput screening (HTS), we adapted our *in vitro* ubiquitination assay to a fluorescence resonance energy transfer (FRET)-based quantification system described previously.^29^ This system uses homogeneous time-resolved fluorescence (HTRF^TM^) to quantify ubiquitin chain reactions. In this system, the fluorescence signals are generated by FRET from two fluorophore-labeled components in proximity, one is ubiquitin and the other is ubiquitinated substrates. In our case, as illustrated in Figure 1b, FRET signals were generated between anti-HA-XL665 that binds to HA-Mdm2 and HA-ubiquitin and ubiquitin cryptate. The total FRET signal from the reaction collectively reflects ubiquitin chains formed on Mdm2 and MdmX. Compounds that disrupt the Mdm2–MdmX interaction will result in reduced E3 ligase activity of Mdm2–MdmX complex consequently reducing the amounts of ubiquitinated Mdm2 and ubiquitinated MdmX and the FRET signals. In the absence of MdmX, FRET signals generated by ubiquitin cryptate and HA-Mdm2 were very low, which was defined as baseline. Under our optimized conditions, addition of MdmX produced ~8-fold increase in FRET signals in an MdmX concentration-dependent manner (Figure 1c) and reaction time-dependent manner (Figure 1d). After adaption of this assay in HT format, we performed an initial screen of ~650 samples. The Z′-factor of this HTS assay was determined to be 0.52 (Figure 1e), indicating a suitable and reliable HTS screen assay (Figure 1e).^30^ This validated HTS assay was then used to screen a diversity library (DIVERSetTM, ChemBridge). Out of 55 230 compounds, we identified a number of positive hits at different inhibition cutoffs as summarized in Figure 1f. The results indicated that our HTS was robust, considering the library size we used and hit rates obtained,^31^ as it identified 119 hits at 90% inhibition cutoff and 371 hits at 70% inhibition cutoff out of ~50 000 compounds (Figure 1f). We followed up all the 371 hits for validation using our bench-top biochemical assay.

### Identification of lead compounds that specifically target Mdm2–MdmX E3 ligase activity

**Figure 2:**
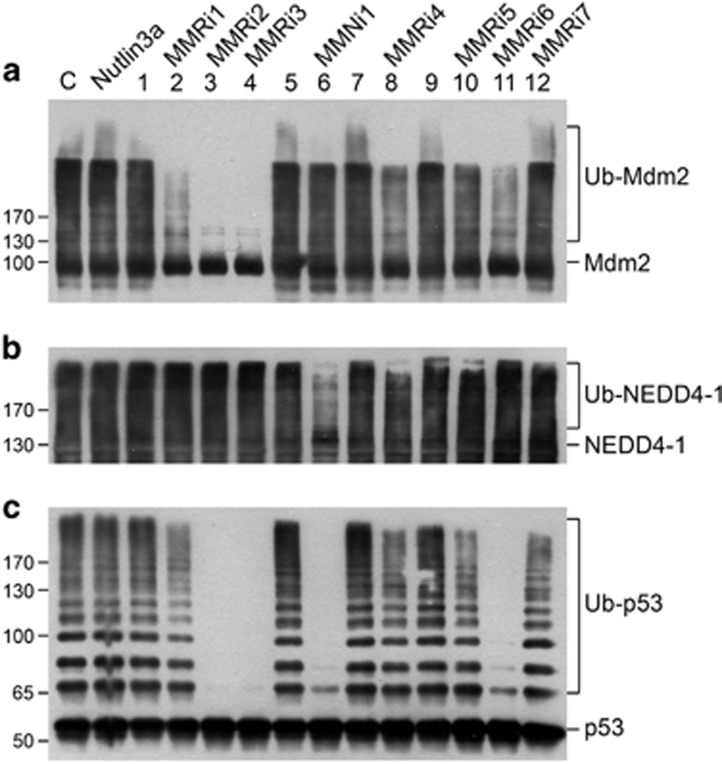
*In vitro* validation of HTS hits by ubiquitination assays. (**a**) Effects of hits on Mdm2 ubiquitination by Mdm2–MdmX complex. Mdm2 (100 nM) and MdmX (200 nM) were used in the *in vitro* ubiquitination reaction in the presence of hits (10 *μ*M) or Nutlin3a (10 *μ*M) or DMSO (10 *μ*M) as a control (C), followed by WB of Mdm2 with anti-HA antibody. Ubiquitinated Mdm2 is shown as Ub-Mdm2. (**b**) Effects of hits on NEDD4-1 autoubiquitination. *In vitro* ubiquitination reaction was carried out with NEDD4-1 (200 nM) as described in (**a**), followed by WB for NEDD4-1 with a rabbit antibody. Ubiquitinated NEDD4-1 is shown as Ub-NEDD4-1. (**c**) Effects of hits on p53 ubiquitination by Mdm2–MdmX. *In vitro* ubiquitination reaction was carried out as described in **a** expect for addition of 200 nM of p53 recombinant proteins, followed by WB of p53 with DO-1 and PAb1801 mixture

To evaluate the hits identified by HTS for specific inhibition of Mdm2–MdmX E3 activity, we examined their ability to inhibit Mdm2 autoubiquitination and p53 polyubiquitination by Mdm2–MdmX using our *in vitro* biochemical assays. We used NEDD4-1 autoubiquitination as a control for nonspecific inhibitors of E3 ligase activity in replicate experiments. In these assays, the ubiquitinated products were monitored by western blotting (WB) instead of FRET assay. After evaluating the available ~350 hits, the hits fall into three categories: (1) 301 hits that fail to inhibit any of the E3 ligase activities (HTS false positive); (2) 42 pan-inhibitors of the ubiquitination system that inhibit all three reactions; (3) seven specific inhibitors of Mdm2–MdmX RING–RING E3 complex that inhibit both MdmX-stimulated Mdm2 autoubiquitination and Mdm2–MdmX-mediated p53 polyubiquitination, but not NEDD4-1 autoubiquitination (designated MMRi, which stands for Mdm2–MdmX RING domain inhibitors). As summarized in Figure 2, among the seven MMRis, three MMRis strongly inhibited p53 ubiquitination by Mdm2–MdmX. We used Nutlin3a as a negative control. In contrast to MMRis, Nutlin3a as an Mdm2–p53-binding inhibitor had no effect on Mdm2–MdmX-mediated p53 polyubiquitination at the same concentration (Figure 2c, Nutlin3a), indicating that MMRis inhibit Mdm2–MdmX via distinct mechanisms than Nutlin3a. Intriguingly, MMRi1, MMRi4 and MMRi6 all partially inhibited Mdm2 autoubiquitination to a similar extent (Figure 2a). However, MMRi6 significantly inhibited p53 ubiquitination, whereas MMRi1 and MMRi4 only partially inhibited p53 ubiquitination by Mdm2–MdmX *in vitro*. The exact mechanisms underlying these differences are unclear at the moment. However, we speculate that these differences may be attributed to the differential effects of the individual MMRis on the ubiquitin transfer preference of Mdm2–MdmX to its adjacent peptides. Of note, this study also identified a group of compounds that inhibits both Mdm2–MdmX and NEDD4-1 E3 ligases designated as MMNi (Mdm2–MdmX and NNEDD4-1 inhibitors) as represented in Figure 2.

### Characterization of MMRi6 and its analogs as disruptors of Mdm2–MdmX RING–RING interaction

**Figure 3:**
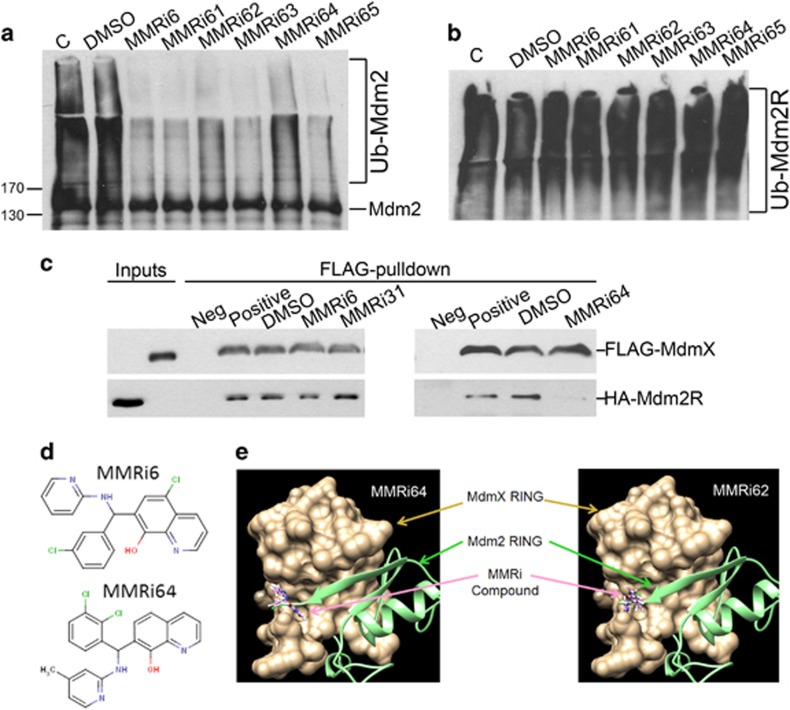
Inhibition of Mdm2–MdmX RING–RING interaction by MMRi6 and its analog MMRi64. (**a**) Effect of MMRi6 and its analogs on the E3 ligase activity of Mdm2–MdmX with Mdm2 autoubiquitination as a readout. *In vitro* ubiquitination assays were performed with Mdm2 (100 nM) and MdmX (200 nM) in the presence of buffer (C), DMSO (10 *μ*M) or indicated compounds (10 *μ*M), followed by WB of Mdm2 with anti-HA antibody. (**b**) Effect of MMRi6 and its analogs on the E3 ligase activity of Mdm2 RING domain. *In vitro* ubiquitination assays were performed with Mdm2 RING domain (100 nM) in the presence of buffer (C), DMSO (10 *μ*M) or indicated compounds (10 *μ*M), followed by WB of Mdm2 with anti-HA. (**c**) Effect of MMRis on interaction of Mdm2 and MdmX proteins *in vitro*. FLAG-MdmX and HA-Mdm2 RING domain (HA-Mdm2R) were incubated *in vitro* in the presence of nothing (positive control), or DMSO (10 *μ*M) or indicated compounds (10 *μ*M), followed by pulldown with anti-FLAG-beads (M2) and WB of MdmX and Mdm2 RING domain with anti-FLAG and anti-HA antibodies respectively. The negative control (Neg) contains all components as in positive control sample except for missing of FLAG-MdmX. (**d**) Chemical structures of MMRi6 and MMRi64. (**e**) Docking analysis of MMRi64 and MMRi62 with 3-D structures of Mdm2–MdmX RING domains. The MMRi62 and MMRi64 (pink) bind to MdmX RING domain (Gold) and interfere with its interaction with Mdm2 RING domain (light green)

MMRi6 was further followed up because several MMRi6 analogs were available and MMRi6 appeared to be the most potent inducer of p53 stabilization in cell culture among the available MMRis (data not shown). Other MMRis of different chemical classes including MMNi will be followed up in separate studies. We obtained 13 commercially available analogs of MMRi6 for further evaluation (structures of MMRi6 and MMRi64 are shown Figure 3d). We confirmed that MMRi6 and its five analogs MMRi61–MMRi65 could effectively inhibit MdmX-stimulated Mdm2 autoubiquitination *in vitro* at 10 *μ*M (Figure 3a). To check whether MMRi6 and its analogs also inhibit autoubiquitination of Mdm2 RING domain, we performed experiments with Mdm2 RING domain recombinant proteins (Figure 3b). Our results indicated that MMRi6 and MMRi61–MMRi65 did not inhibit autoubiquitination of Mdm2 RING domain at equimolar concentrations, suggesting that these compounds selectively affect Mdm2–MdmX RING–RING interaction but not Mdm2–Mdm2 RING–RING interaction. To directly evaluate the ability of these compounds in inhibiting Mdm2–MdmX interaction, we performed *in vitro* pulldown experiments using recombinant FLAG-MdmX and HA-Mdm2 RING proteins. After incubation of the two proteins in the presence or absence of compounds, FLAG-MdmX was pulled down with anti-FLAG beads. The MdmX-bound HA-Mdm2 RING domain was then detected for HA-tag through WB analysis. Our results indicated that MMRi6 and MMRi64 effectively inhibited Mdm2–MdmX interaction *in vitro*. In contrast, MMRi31, an analog of MMRi3 strongly inhibited E3 ligase activity but not the interaction of Mdm2–MdmX. Rather, it slightly increased the interaction (Figure 3c). Furthermore, our docking analysis using the DOCK6 program and the 3-D structure of Mdm2–MdmX RING domains^32^ indicated that MMRi62 and MMRi64 bind to the MdmX RING domain (Figure 3e, gold color). Their binding to the MdmX cleft interferes with the interactions between MdmX RING domain and Mdm2 RING domain (Figure 3e, green color). Collectively, these results confirmed that MMRi6 and MMRi64 are disrupting inhibitors of Mdm2–MdmX RING domain interaction.

### Activation of the p53 pathway by MMRi

**Figure 4:**
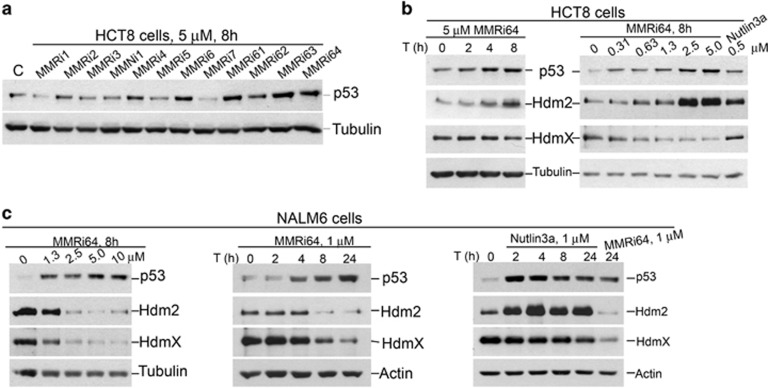
MMRis activate the p53 pathway in cancer cell lines. (**a**) p53 protein accumulation in MMRi-treated HCT-8 cells. HCT-8 cells were treated with indicated compounds (5 *μ*M) for 8 h and the whole-cell lysates were analyzed by WB of p53 with tubulin as a loading control. (**b**) Time and concentration-dependent induction of p53 and Mdm2 accumulation by MMRi64 in HCT-8 cells. HCT-8 cells were treated at the indicated concentrations of MMRi64 (right) for 8 h and for the indicated time at 5 *μ*M (left) and whole-cell lysates were analyzed by WB of HdmX, Hdm2 and p53. Tubulin was used for loading control. (**c**) Activation of the p53 pathway by MMRi64 in NALM6 cells. NALM6 cells were treated for indicated time at the indicated concentrations of MMRi64 or Nutlin3a and the whole-cell lysates were analyzed by WB for p53, Hdm2, HdmX and tubulin

Using HCT-8, a wild-type (WT) p53-bearing colon cancer cell line, we tested the activity of our MMRis in activating the p53 pathway. MMRi6 and its analogs appeared to be the most potent inducers of p53 protein stabilization (Figure 4a). Among the analogs, MMRi64 was further followed up for more cellular experiments. In HCT-8 cells, MMRi64 at 5 *μ*M induced a time-dependent p53 accumulation accompanied with induction of its target gene product Mdm2 (Figure 4b, left panel). MMRi64 also induced a concentration-dependent induction of p53 accumulation, which was evident at a concentration as low as 0.31 *μ*M (Figure 4b, right panel). Interestingly, MMRi64 induced significant downregulation of MdmX in a time-dependent and concentration-dependent manner (Figure 4b), which was not observed with Mdm2–p53 inhibitor Nutlin3a (Figure 4b, right panel). To further test whether MMRi64 also activates p53 in other cancer types, we performed experiments with pre-B acute lymphoblastic leukemia NALM6 cells that bear WT p53. As indicated in Figure 4c, MMRi64 was capable of activating p53 in NALM6 cells as well in both time-and-concentration-dependent manners. Surprisingly, in contrast to HCT-8 cells, Mdm2 expression was strongly reduced by MMRi64 in NALM6 cells, in addition to MdmX downregulation. We performed a similar experiment with Nutlin3a. As shown in Figure 4c left panel, Nutlin3a strongly induced the levels of Mdm2 protein and slightly decreased MdmX levels. These data indicated that MMRi64 has unique inhibitory effect on Mdm2 and MdmX expression levels in leukemia cells.

### MMRi64 potently induces p53-dependent and p53-independent apoptosis in lymphoma cells

**Figure 5:**
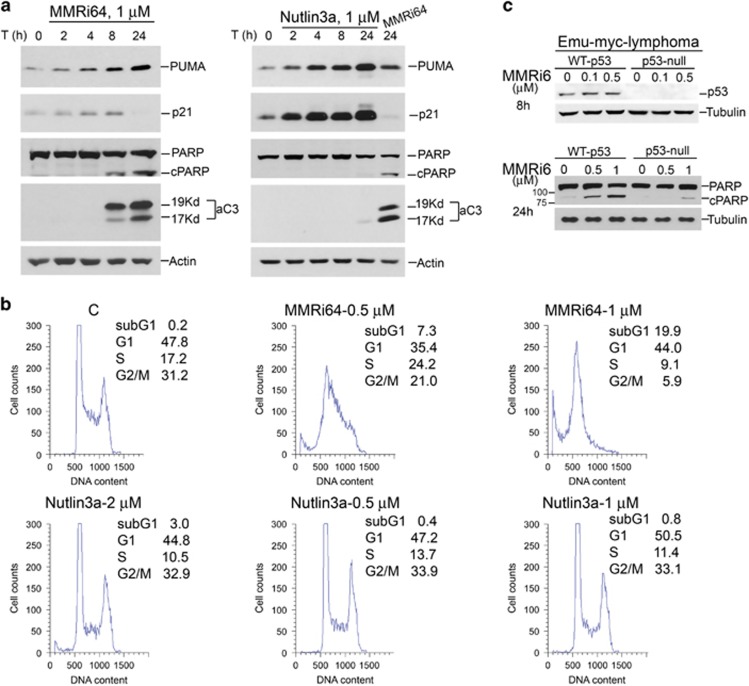
MMRi64 preferentially induces apoptosis in lymphoma cells. (**a**) MMRi64 is better inducer of apoptotic response than Nutlin3a in NALM6 cells. Whole-cell lysates were prepared from time course treatment of NALM6 cells with 1 *μ*M of MMRi64 (left) or Nutlin3a (right) and followed by WB for PUMA, p21, PARP, active caspase 3 (aC3) and actin. (**b**) MMRi64 more effectively induces apoptosis revealed by flow cytometry. Equimolar concentration of MMRi64 and Nutlin3a (1 *μ*M) was used to treat NALM6 cells for 24 h followed by flow cytometry analysis after fixation and PI-staining of the cells. Sub-G1 fractions of each treatment were shown. The data were from triplicates of a single experiment with standard deviations within 0.06–0.5%. (**c**) Effects of p53 status on MMRi6-induced PARP cleavage in Emu-myc lymphoma cells. The Emu-myc lymphoma cells of different p53 status were treated with 0.1 and 0.5 *μ*M of MMRi6 for 8 h and whole lysates were subjected to WB for p53 (upper panel); or the cells were treated with 0.5 and 1 *μ*M of MMRi64 for 24 h for WB of PARP with tubulin serving as loading control

We further tested the antitumor effect of MMRi64 in leukemia/lymphoma cells. We focused on the induction of apoptosis by this compound because p53-dependent apoptosis is a critical mechanism for preventing lymphomagenesis ^33, 34^ and determines the outcome of lymphoma treatment.^35^ In NALM6 cells, MMRi64 at 1 *μ*M showed a time-dependent induction of PUMA, a critical pro-apoptotic downstream gene product of p53.^36, 37^ Interestingly, p21, the growth-arresting effector target gene of p53, was transiently induced then downregulated to a level lower than basal p21 expression at 24 h of the treatment. Accompanied with the activation of p53's pro-apoptotic arm of the p53 response, cleavage of poly ADP ribose polymerase (PARP) by activated caspase 3 was evident at 8 h and further increased at 24 h after treatment. These data indicated that MMRi64 triggered activation of the intrinsic apoptosis pathway. We performed a similar experiment with Nutlin3a and our results showed that Nutlin3a induced stronger p53 accumulation and PUMA induction than MMRi64 at the same concentration of MMRi64 (Figure 5a, middle panel). However, in contrast to MMRi64, Nutlin3a also exhibited stronger expression of growth-arresting effector p21 (Figure 5a, right panel). Accompanied with these molecular events, cleavage of PARP and activation of caspase 3 were barely detectable in Nutlin3a-treated cells for 24 h, although Nutlin3a induced a similar level of p53 accumulation at this time point (Figure 5a right panel). Together, these results indicated that p53 activation by Nutlin3a mainly results in cell growth arrest, whereas p53 activation by MMRi64 mainly causes apoptosis in NALM6 cells. To determine whether MMRi64-induced apoptosis in lymphoma cells is p53 dependent, we performed experiments with Emu-myc mouse lymphoma cells of different *p53* status. As expected, MMRi6 induced p53 accumulation in wt-p53 cells at a concentration as low as 0.1 *μ*M (Figure 5b, upper panel). PARP cleavage was detected at 24 h by ~0.5 *μ*M MMRi6 treatment in wt-p53 Emu-myc lymphoma cells but not in p53-null Emu-myc lymphoma cells (Figure 5b, lower panel). Therefore, the MMRi6-induced apoptosis in lymphoma cells contains a p53-dependent component. The ability of MMRi64 to induce apoptosis was further analyzed by flow cytometry. Our results showed that MMRi64 at 0.5 and 1 *μ*M for 48 h induced 7.3% and 20% sub-G1 population, respectively. In contrast, Nutlin3a at 0.5, 1 and 2 *μ*M for 48 h only induced 0.4%, 0.8% and 3.0% sub-G1 populations, respectively (Figure 5c). Together, these results indicate that MMRi64 preferentially induces apoptosis in NALM6 cells.

**Figure 6:**
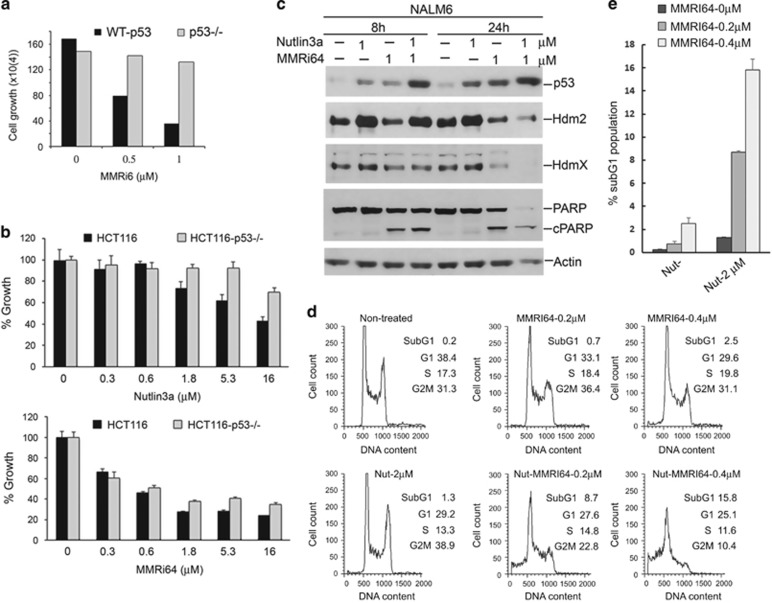
MMRi64 lymphoma cell growth via p53-dependent and p53-independent mechanisms. (**a**) Effects of p53 status on MMRi64-induced growth inhibition in Emu-myc lymphoma cells. The mouse Emu-myc lymphoma cells of different p53 status were cultured in the presence of 0.5 and 1 *μ*M of MMRi64 and the numbers of viable cells were counted by Trypan blue exclusion at 72 h of treatment and plotted in histograms. (**b**) Effect of p53 status on Nutlin3a and MMRi64 sensitivity in colon cancer cells. HCT116 and HCT116-p53-/- cells were treated with indicated concentrations of drugs for 72 h and drug-induced growth inhibition was measured by MTT method and plotted in histograms. (**c**) Effect of MMRi64-Nutlin3a combination on expression of p53, Mdm2, MdmX and apoptotic cleavage of PARP in NALM6 cells. WB analysis of p53, Mdm2, MdmX and PARP cleavage after NALM6 cells were treated with Nutlin3a (1 *μ*M) or MMRi64 (1 *μ*M) alone or in combination for 8 and 24 h. (**d**) Flow cytometric analysis of NALM6 cells treated with indicated concentrations of Nutlin3a or MMRi64 alone or in combination for 48 h. Cells were fixed and stained with PI and subjected to flow cytometric analysis. The data were from triplicates of a single experiment with standard deviations within 0.06–0.5%. (**e**) Histograms of sub-G1 populations induced by MMRi64 or Nutlin3a alone or in their combinations at the indicated concentrations

To further confirm the p53 dependence of MMRi64-induced growth inhibition, we used mouse Emu-myc lymphoma cells of wt-p53 and p53-null background in growth inhibition experiments. A 72-h cell proliferation assay for MMRi6 showed IC50s of ~0.5 *μ*M and ~3 *μ*M in wt-p53 and p53-null Emu-myc lymphoma cells, respectively, indicating that p53 contributes to a ~6-fold difference in MMRi6 sensitivity in this set of mouse lymphoma cells (Figure 6a, data from two doses were shown). Furthermore, we used HCT116 and HCT116-p53−/− cells to test the contribution of p53 to MMRi64-induced anti-growth effect. As shown in Figure 6b, at equimolar concentrations of Nutlin3a, p53 contributes to a maximal ~35% more growth inhibition than that of HCT116-p53−/− cells, whereas p53 contributes to a maximal ~10% more growth inhibition in MMRi64 treatment. Therefore, MMRi64 inhibits cell growth through both p53-dependent and p53-independent mechanisms.

To understand whether low concentrations of MMRi64 and Nutlin3a will synergistically inhibit cell growth by apoptosis, we performed combination experiments with the two compounds in NALM6 cells. First, we looked at apoptotic PARP cleavage during single and combination treatment. Our results showed that 1 *μ*M Nutlin3a and 1 *μ*M MMRi64 induced similar levels of p53 accumulation at 8 and 24 h. However, only MMRi64 induced obvious PARP cleavage at 8 and 24 h. Yet, combination of the two compounds markedly induced p53 and PARP cleavage at the two time points. These results were consistent with sub-G1 population analysis by flow cytometry. Single treatment at low concentrations with either Nutlin3a or MMRi64 induced small increase in sub-G1 populations (0.73% and 2.5% for 0.2 *μ*M or 0.4 *μ*M of MMRi64, respectively, and 1.3% for 2 *μ*M Nutlin3a). As expected, the combination of Nutlin3a-MMRi64 at two different concentrations caused a significant increase in sub-G1 populations: 8.7% for 2 *μ*M Nutlin3a-0.2 *μ*M MMRi64 combination and 16% for 2 *μ*M Nutlin3a-0.4 *μ*M MMRi64 combination. Overall, these results indicate that combinations of MMRi64 and Nutlin3a synergistically kill lymphoma cells by apoptosis.

## Discussion

Targeting Mdm2–p53 interaction for p53-based cancer therapy has been pursued for many years. Several promising compounds with therapeutic activity in preclinical systems have been reported and have recently advanced into early clinical trials.^2^ Nutlin3a was the first potent specific inhibitor of Mdm2–p53 interaction.^1^ It has served as a prototype for chemical optimization and has fostered the discovery of compounds with better drug properties and efficacy.^38, 39, 40^ However, one prominent concern is that MdmX overexpression confers resistance to Nutlin3a.^14, 15, 16^ This problem is likely to hinder the use of other Mdm2–p53 targeting compounds. Together with the radioresistant lymphoma phenotype of non-degradable MdmX mutant mice,^41^ these findings point MdmX as another valid drug target for p53-based cancer therapy.^13^ Using an elegant mouse model, Even's group demonstrated that MdmX is a better drug target than Mdm2 in lymphoma.^42^ To overcome MdmX-mediated resistance, dual inhibitors that target both Mdm2–p53 and MdmX–p53 interfaces were also explored. Results from a dual inhibitor peptides and compounds demonstrated better p53-dependent cytotoxic effects in breast and colon cancer cells.^17, 43^

Differing from the focus of the field, we turned our interest in Mdm2–MdmX RING–RING interaction based on the biochemical findings from our lab and genetic evidences from other's studies.^23, 24, 26^ This report is the first attempt to identify specific inhibitors of Mdm2–MdmX RING domain interaction and assess the effectiveness of targeting this newly established interface. To our surprise, MMRi64 not only distuptedMdm2–MdmX interaction, but also induced a significant MdmX downregulation in leukemia cells. Mdm2 upregulation usually follows p53 activation because of a feedback regulatory loop.^44, 45^ However, Mdm2 was not induced in MMRi64-treated leukemia cells (Figure 4c), although it was induced in HCT-8 colon cancer cells (Figure 4b). The mechanisms underlying the cell type difference in Mdm2 induction are not known at present. We speculate that this may be due to the differential effect of MdmX on Mdm2 protein stability, that is, MdmX has a crucial role to stabilize Mdm2 proteins in leukemia cells but is not so critical in colon cancer cells. This hypothesis needs further experimentation. In our opinion, this cell type-specific effect of MMRi64 on Mdm2 and MdmX makes it a very unique compound for leukemia/lymphoma treatment.

We tested MMRi64 mainly in leukemia/lymphoma cells because the p53 pathway is critical for apoptosis induction,^46^ lymphoma development^10, 33, 34^ and restoration of p53 in mice leads to regression of autochthonous lymphomas.^4^ Importantly, p53 mutation rate is relatively low (4.2% of diffuse large B-cell lymphoma, TCGA data) and about 80–90% of lymphoma patients have a wt-p53 status.^47^ Therefore, p53-based therapy would benefit a large group of lymphoma/leukemia patients. The p53-based therapy relies on drug-induced apoptosis.^4^ In sharp contrast to Nutlin3a, MMRi64 preferentially induces apoptosis in leukemia/lymphoma cells, probably due to PUMA induction and simultaneous shutdown of pro-growth-arrest p21 (Figures 4 and 5a), and downregulation of Mdm2 and MdmX. Whether the selective effect of p53 downstream gene induction by MMRi64 is due to its effect on MdmX or Mdm2 needs to be tested in future experiments.

The cellular effect of Nutlin3a is very moderate at concentrations similar or higher than that of MMRi64: 1–3% increase in G1 or G2 populations and 4–7% reduction in S-phase cells (Figure 5b), suggesting that there are modifiers of the p53 downstream effect in NALM6 cells. The mechanisms to why MMRi64 preferentially induces apoptosis compared with Nutlin3a are presently unknown. Downregualtion of Mdm2 and/or MdmX by MMRi64 may be an explanation and several lines of evidence support this notion. The downregulation of Mdm2 by MI-219 appears to be associated with p53-dependent apoptosis in follicular lymphoma.^48^ Lozano's group demonstrated that p53 restoration in Mdm2-overexpressing tumors inhibits proliferation but does not induce apoptosis,^49^ suggesting that high levels of Mdm2 has anti-death activity. Moreover, siRNA knockdown experiments revealed that Mdm2 is actually required for p53-dependent induction of p21 to cause growth arrest.^50^ These findings are consistent with the reduced levels of Mdm2 and weak induction of p21 by MMRi64 treatment. Shutdown of p21 induction by p53 in MMRi64-treated cells is expected to favor apoptosis induction in MMRi64-treated cells, as p21 serves as an inhibitory effector for p53-dependent apoptosis shown in colon cancer cells.^51^ In addition, Mdm2 was reported to have p53-independent lymphomagenic activity, a role revealed by Mdm2 splice isoforms that do not bind to p53 but promote Emu-myc lymphomagenesis in a manner comparable with full-length Mdm2.^52^ At present, we cannot exclude the possibility that MMRi64 kills leukemia/lymphoma cells independently of p53 or PUMA, which requires more mechanistic studies in the future. The mechanisms underlying downregulation of MdmX and Mdm2 in the presence of MMRi64 in cells are not clear either. However, we speculate that the binding of MMRi64 to MdmX disrupts Mdm2–MdmX interaction, which may make MdmX a target of protein quality control system leading to its ubiquitin-dependent degradation. This is highly likely as compound-bound MdmX may adopt a conformation of ill-folded protein, which will be recognized by protein quality control E3 ligase CHIP. Once MdmX is degraded, it will affect Mdm2 expression as MdmX is required for binding p53 to Mdm2 promoter and for the full induction of Mdm2 in stressed cells.^53^ In addition, MdmX can stabilize Mdm2 protein by inhibiting its autoubiquitination.^54^ Of note, MEL23 and MEL24 were identified by a cell-based Mdm2-luciferase fusion reporter screening effort as inhibitors of Mdm2–MdmX E3 ligase.^55^ However, their mechanisms of action were not characterized and their potential in apoptosis induction was not reported. As MEL23/MEL24 strongly stabilize Mdm2 and MdmX,^55^ our MMRi64 has unique property for better targeting oncogenic Mdm2–MdmX complex for robust cancer cell killing.

MMRi64 belongs to a chemical class of quinolinol family in which inhibitors of botulinum neurotoxins and two anticancer derivatives were recently reported.^56, 57, 58^ Owing to the unique effects of MMRi64 on the critical components of the p53 regulatory loop and downstream effectors, MMRi64 may be used as a pharmaceutical tool to dissect the molecular regulation of p53-dependent transactivation program. Therefore, this study identified a new class of chemicals that may be useful in basic research on p53-dependent biology as well as development of new p53/Mdm2/MdmX-based cancer therapeutics. In summary, MMRi represents a new class of p53-activating agents with promising anticancer activities in cell culture. Future studies will be needed to address the biophysical property of drug–target interaction, p53/Mdm2/MdmX–dependency and the issues of off-targets and genotoxicity in lead optimization process.

## Materials and Methods

### Plasmids, protein purification and chemical reagents

FLAG-MdmX and HA-Mdm2 (human) constructs for insect cell expression and protein purification were described previously.^26^ HA-ubiquitin construct was generated by inserting HA-tag to the N-terminus of ubiquitin in pET28a vector. The mammalian expression plasmid pcDNA3.1-HdmX was a gift from Dr. Gokul Das (Roswell Park Cancer Institute). HA-Mdm2 RING domain was generated by site-directed mutagenesis to loop out aa28-299 using pFAST-bac-HA-Mdm2 as an template and the recombinant baculovirus was prepared and protein was expressed in insect cells as described previously.^26^

HCT-8 was used in our recent studies^59^ and originally purchased from ATCC (Manassas, VA, USA) and were maintained in Dulbecco's modified Eagle's medium (DMEM) supplemented with 10% fetal calf serum (FCS; Atlanta Biologicals, Inc., Flowery Branch, GA, USA) and antibiotics. HCT116 and HCT116-p53−/− cells were originally provided to Dr. Terry Beerman by Professor B Vogelstein (Johns Hopkins University, Baltimore, MD, USA). These cells were received in 2004 and cultured in McCoy's 5A containing 10% fetal bovine serum in an atmosphere of 5% CO_2_. Pre-B acute lymphoblastic leukemia cell line NALM6 cells^60^ were obtained from Fengzhi Li (RPCI) and cultured in RPMI-1640 supplemented with 10% FCS (Atlanta Biologicals, Inc.) and antibiotics. Emu-myc lymphoma cells of wt-p53 and p53-null background were kind gifts from Dr. Scott Lowe and Dr. Clare Scott, respectively. These cells were cultured in the high-glucose version of DMEM supplemented with 10% FCS, penicillin/streptomycin, 0.1 mM l-asparagine and 50 *μ*M 2-mercaptoethanol.

Lead compounds were purchased from Hit2lead Chembridge Online Chemical Store (San Diego, CA, USA). All the lead compounds were dissolved in DSMO as 20 mM stock. Nutlin3a was purchased from Cayman Chemical Company (Ann Arbor, MI, USA). Antibodies for p53 (DO-1 and 1801) and p21 (H-164) were purchased from Santa Cruz (Dallas, TX, USA). Rabbit antibodies against PUMA (D30C10), Bcl-2 (50E3), PARP (46011D) and cleaved caspase 3 (D175) were purchased from Cell Signaling Technology (Danvers, MA, USA). Monoclonal antibodies for Mdm2 (2A9 and 4B11) were kind gifts from Dr. Moshe Oren (Weizmann Institute of Science, Rehovot, Israel). The rabbit polyclonal antibody for MdmX was purchased from Proteintech (Chicago, IL, USA) (cat #17914-1-AP). Anti-FLAG was from Sigma (St Louis, MO, USA) (M2, F1804)) and anti-HA (HA.11) was from Covance (Princeton, NJ, USA). Anti-HA-XL665 (610HAXLB) and ubiquitin cryptate (61UBIKLB) were purchased from Cisbio (Bedford, MA, USA) and reconstituted according to the manufacturer's instruction.

### FRET-based *in vitro* ubiquitination assay

FRET-based *in vitro* ubiquitination assay was adapted from the protocol of HTRF^TM^ described previously.^29^ HTRF is a homogeneous method, which combines standard FRET technology with time-resolved measurement of fluorescence. The HTRF^TM^ emission were measured at two different wavelengths, 615 nm (donor) and 665 nm (acceptor). In the MdmX-stimulated Mdm2 autoubiquitination reaction, the ubiquitin cryptate and HA-tagged ubiquitin were incorporated into the ubiquitin chain. FRET is generated when the XL665 labeled anti-HA antibody binds to the HA-tagged Hdm2 or HA-tagged ubiquitin. The amounts of each reagent and recombinant protein were optimized before being adapted to high-throughput (HT) format with concoction for two pre-mixtures for convenient handling by a robot hand.

### HTS with the method of HTRF^TM^

The pre-reaction mixture one consisted of 40 mM Tris-HCl (pH 7.5), 5 mM MgCl_2_, 2 mM DTT, 5 mM ATP, 20 nM E1, 350 nM E2 (UbcH5), 25 nM HA-tagged Mdm2, 200 nM MdmX. First, 10 *μ*l of the pre-reaction one was dispensed in each well of 384-well plate (Multiflo, Biotek, Winooski, VT, USA). Then compounds from a chemical library (DIVERSet, ChemBridge) were added in a volume of 8 nl of each by the robot pin tool (PerkinElmer JANUS, Waltham, MA, USA, V&P Scientific 384 Pin tool, San Diego, CA, USA). The reaction was started by adding premixture two, which consisted of 250 nM HA-tagged ubiquitin and 50 nM ubiquitin cryptate at 2 *μ*l per well. After incubation at 37 °C for 1.5 h, the reaction was terminated by adding 10 *μ*l of the detection buffer, which contains 50 mM phosphate buffer pH 7.0, 0.1% BSA, 0.1 M EDTA, 0.8 M KF and 20 nM XL665-conjugated antibody against HA tag. The reaction was kept for 1 h at room temperature before measuring the FRET signal. For the FRET measurement in Perkin Elmer Envision 2103 Multilabel Reader, there is a 100 *μ*s time delay between the excitation (320 nm) and measurement at two different wavelengths (615  and 665 nm), then calculating the ratio for each well individually (ratio=665 nm/615 nm x10^4^). The 10^4^ multiplying factor is introduced for convenient data processing.

### *In vitro* validation assays by Mdm2 autoubiquitination and p53 ubiquitination by Mdm2–MdmX and NEDD4-1 autoubiquitination

In order to test the compound specificity, we used two sets of ubiquitination reaction. One was MdmX-stimulated Mdm2 autoubiquitination, the other was NEDD4-1 autoubiquitination. The two reactions shared the same constitutes in the premixture: 40 mM Tris-HCl (pH 7.5), 5 mM MgCl_2_, 2 mM DTT, 5 mM ATP, 20 nM E1 and 350 nM E2 UbcH5c and 10 μM of ubiquitin. The former reaction included 100 nM HA-tagged Mdm2 and 200 nM MdmX, and latter reaction included 200 nM HA-tagged NEDD4-1. After adding compound to a final concentration of 10 μM, the reaction was started by incubation at 30 °C in a water bath for 1 h. Then the reaction was stopped by adding SDS sample buffer, followed by SDS-PAGE and WB analysis for HA or NEDD4-1. *In vitro* assays for p53 ubiquitination by Mdm2–MdmX were performed as described previously.^26^ Briefly, reactions were carried out as described above for Mdm2 autoubiquitination except for addition of 100 nM p53. Compounds in dimethyl sulfoxide (DMSO) or DMSO of final concentrations of 10 μM were added in the reaction before starting the reaction at 30 °C for 1 h followed by WB of p53 with DO-1 antibody.

### Proliferation and apoptosis assays

Growth inhibition assay and apoptosis assay was described previously.^59^

### RING domain interaction between Mdm2 and MdmX by *in vitro* pulldown assay

HA-tagged Mdm2 RING domain (500 nM), Flag-tagged MdmX (250 nM) and testing compound (10 *μ*M) were mixed together in 50 μl NP40 buffer (0.5% NP40, 150 mM NaCl, 20 mM Tris-HCl, pH 8.0). After 30-min incubation, the protein mixture was diluted with 450 μl NP40 buffer-0.5% BSA. In all, 10 *μ*l of anti-FLAG antibody conjugated M2 beads (Sigma: A2220) preincubated with NP40 buffer containing 0.5% BSA for 30 min at RT were added into the mixture to pulldown the Flag-MdmX by rotating at RT for 2 h. After washing five times by the NP40 buffer-0.5% BSA, the M2 beads were eluted with 45 *μ*l 0.2 mg/ml 3xFlag peptides (in 20 mM Tris-HCl, pH 7.5, 10 mM NaCl, 1 mM DTT) to release FLAG-MdmX and its interacting proteins. The FLAG-MdmX-bound HA-Mdm2 RING was detected by SDS-PAGE followed by WB analysis for HA-Mdm2 by using ant-HA antibody.

### Docking analysis

Docking studies were carried out in order to investigate the preferential binding mode geometry of the compounds. The steps were performed as instructed by DOCK6 software package (dock.compbio.ecsf.edu) including all default parameter set. 3-D grids were defined for the interaction with the compounds. Docking results were examined using the Chimera program (http://www.cgl.ucsf.edu/chimera/) to observe the interaction precisely at atomic level. Chimera was also used to manipulate the Mdm2–MdmX RING (30) structures with the utilities for deleting solvents and adding charges.^32^

## Abbreviations

DMSO: dimethyl sulfoxide
FCS: fetal calf serum
FRET: fluorescence resonance energy transfer
GST: glutathione *S*-transferase
HT: high-throughput
HTS: high-throughput screening
MDM2: mouse double minute 2
MDMX: (MDM4), mouse double minute 4
MMNi: MdmX and NEDD4-1 inhibitors
MMRi: Mdm2–MdmX RING domain inhibitors
NEDD4: neural precursor cell expressed developmentally downregulated protein 4
PARP: poly ADP ribose polymerase
PUMA: p53 upregulated modulator of apoptosis
RB: retinoblastoma protein
RING: really interesting new gene
RING–RING interaction: RING domain–RING domain interaction
WB: western blotting
WT: wild type

## References

[bib1] 1Vassilev LT, Vu BT, Graves B, Carvajal D, Podlaski F, Filipovic Z et al. *In vivo* activation of the p53 pathway by small-molecule antagonists of MDM2. Science 2004; 303: 844–848. 14704432 10.1126/science.1092472

[bib2] 2Khoo KH, Verma CS, Lane DP. Drugging the p53 pathway: understanding the route to clinical efficacy. Nat Rev Drug Discov 2014; 13: 217–236. 24577402 10.1038/nrd4236

[bib3] 3Martins CP, Brown-Swigart L, Evan GI. Modeling the therapeutic efficacy of p53 restoration in tumors. Cell 2006; 127: 1323–1334. 17182091 10.1016/j.cell.2006.12.007

[bib4] 4Ventura A, Kirsch DG, McLaughlin ME, Tuveson DA, Grimm J, Lintault L et al. Restoration of p53 function leads to tumour regression *in vivo* Nature 2007; 445: 661–665. 17251932 10.1038/nature05541

[bib5] 5Xue W, Zender L, Miething C, Dickins RA, Hernando E, Krizhanovsky V et al. Senescence and tumour clearance is triggered by p53 restoration in murine liver carcinomas. Nature 2007; 445: 656–660. 17251933 10.1038/nature05529 PMC4601097

[bib6] 6Levine AJ, Oren M. The first 30 years of p53: growing ever more complex. Nat Rev Cancer 2009; 9: 749–758. 19776744 10.1038/nrc2723 PMC2771725

[bib7] 7Berglind H, Pawitan Y, Kato S, Ishioka C, Soussi T. Analysis of p53 mutation status in human cancer cell lines: a paradigm for cell line cross-contamination. Cancer Biol Ther 2008; 7: 699–708. 18277095 10.4161/cbt.7.5.5712

[bib8] 8Howes KA, Ransom N, Papermaster DS, Lasudry JG, Albert DM, Windle JJ. Apoptosis or retinoblastoma: alternative fates of photoreceptors expressing the HPV-16 E7 gene in the presence or absence of p53. Genes Dev 1994; 8: 1300–1310. 7986270 10.1101/gad.8.11.1300

[bib9] 9Cui H, Schroering A, Ding HF. p53 mediates DNA damaging drug-induced apoptosis through a caspase-9-dependent pathway in SH-SY5Y neuroblastoma cells. Mol Cancer Ther 2002; 1: 679–686. 12479364

[bib10] 10Hemann MT, Zilfou JT, Zhao Z, Burgess DJ, Hannon GJ, Lowe SW. Suppression of tumorigenesis by the p53 target PUMA. Proc Natl Acad Sci USA 2004; 101: 9333–9338. 15192153 10.1073/pnas.0403286101 PMC438977

[bib11] 11Laurie NA, Donovan SL, Shih CS, Zhang J, Mills N, Fuller C et al. Inactivation of the p53 pathway in retinoblastoma. Nature 2006; 444: 61–66. 17080083 10.1038/nature05194

[bib12] 12Li Q, Lozano G. Molecular pathways: targeting Mdm2 and Mdm4 in cancer therapy. Clin Cancer Res 2013; 19: 34–41. 23262034 10.1158/1078-0432.CCR-12-0053 PMC3537867

[bib13] 13Wade M, Wahl GM. Targeting Mdm2 and Mdmx in cancer therapy: better living through medicinal chemistry? Mol Cancer Res 2009; 7: 1–11. 19147532 10.1158/1541-7786.MCR-08-0423 PMC2629357

[bib14] 14Patton JT, Mayo LD, Singhi AD, Gudkov AV, Stark GR, Jackson MW. Levels of HdmX expression dictate the sensitivity of normal and transformed cells to Nutlin-3. Cancer Res 2006; 66: 3169–3176. 16540668 10.1158/0008-5472.CAN-05-3832

[bib15] 15Hu B, Gilkes DM, Farooqi B, Sebti SM, Chen J. MDMX overexpression prevents p53 activation by the MDM2 inhibitor Nutlin. J Biol Chem 2006; 281: 33030–33035. 16905541 10.1074/jbc.C600147200

[bib16] 16Gembarska A, Luciani F, Fedele C, Russell EA, Dewaele M, Villar S et al. MDM4 is a key therapeutic target in cutaneous melanoma. Nat Med 2012; 18: 1239–1247. 22820643 10.1038/nm.2863 PMC3744207

[bib17] 17Hu B, Gilkes DM, Chen J. Efficient p53 activation and apoptosis by simultaneous disruption of binding to MDM2 and MDMX. Cancer Res 2007; 67: 8810–8817. 17875722 10.1158/0008-5472.CAN-07-1140

[bib18] 18Chang YS, Graves B, Guerlavais V, Tovar C, Packman K, To KH et al. Stapled alpha-helical peptide drug development: a potent dual inhibitor of MDM2 and MDMX for p53-dependent cancer therapy. Proc Natl Acad Sci USA 2013; 110: E3445–E3454. 23946421 10.1073/pnas.1303002110 PMC3767549

[bib19] 19Sdek P, Ying H, Chang DL, Qiu W, Zheng H, Touitou R et al. MDM2 promotes proteasome-dependent ubiquitin-independent degradation of retinoblastoma protein. Mol Cell 2005; 20: 699–708. 16337594 10.1016/j.molcel.2005.10.017

[bib20] 20Jin Y, Zeng SX, Sun XX, Lee H, Blattner C, Xiao Z et al. MDMX promotes proteasomal turnover of p21 at G1 and early S phases independently of, but in cooperation with, MDM2. Mol Cell Biol 2008; 28: 1218–1229. 18086887 10.1128/MCB.01198-07 PMC2258738

[bib21] 21Tang J, Qu L, Pang M, Yang X. Daxx is reciprocally regulated by Mdm2 and Hausp. Biochem Biophys Res Commun 2010; 393: 542–545. 20153724 10.1016/j.bbrc.2010.02.051 PMC2838978

[bib22] 22Itahana K, Mao H, Jin A, Itahana Y, Clegg HV, Lindstrom MS et al. Targeted inactivation of Mdm2 RING finger E3 ubiquitin ligase activity in the mouse reveals mechanistic insights into p53 regulation. Cancer Cell 2007; 12: 355–366. 17936560 10.1016/j.ccr.2007.09.007

[bib23] 23Pant V, Xiong S, Iwakuma T, Quintas-Cardama A, Lozano G. Heterodimerization of Mdm2 and Mdm4 is critical for regulating p53 activity during embryogenesis but dispensable for p53 and Mdm2 stability. Proc Natl Acad Sci USA 2011; 108: 11995–12000. 21730132 10.1073/pnas.1102241108 PMC3141986

[bib24] 24Huang L, Yan Z, Liao X, Li Y, Yang J, Wang ZG et al. The p53 inhibitors MDM2/MDMX complex is required for control of p53 activity *in vivo*. Proc Natl Acad Sci USA 2011; 108: 12001–12006. 21730163 10.1073/pnas.1102309108 PMC3141917

[bib25] 25Linares LK, Hengstermann A, Ciechanover A, Muller S, Scheffner M. HdmX stimulates Hdm2-mediated ubiquitination and degradation of p53. Proc Natl Acad Sci USA 2003; 100: 12009–12014. 14507994 10.1073/pnas.2030930100 PMC218704

[bib26] 26Wang X, Wang J, Jiang X. MdmX protein is essential for Mdm2 protein-mediated p53 polyubiquitination. J Biol Chem 2011; 286: 23725–23734. 21572037 10.1074/jbc.M110.213868 PMC3129153

[bib27] 27Wang X, Jiang X. Mdm2 and MdmX partner to regulate p53. FEBS Lett 2012; 586: 1390–1396. 22673503 10.1016/j.febslet.2012.02.049

[bib28] 28Landre V, Rotblat B, Melino S, Bernassola F, Melino G. Screening for E3-ubiquitin ligase inhibitors: challenges and opportunities. Oncotarget 2014; 5: 7988–8013. 25237759 10.18632/oncotarget.2431 PMC4226663

[bib29] 29Yabuki N, Watanabe S, Kudoh T, Nihira S, Miyamato C. Application of homogeneous time-resolved fluorescence (HTRFTM) to monitor poly-ubiquitination of wild-type p53. Comb Chem High Throughput Screen 1999; 2: 279–287. 10539989

[bib30] 30Zhang JH, Chung TD, Oldenburg KR. A simple statistical parameter for use in evaluation and validation of high throughput screening assays. J Biomol Screen 1999; 4: 67–73. 10838414 10.1177/108705719900400206

[bib31] 31Hughes JP, Rees S, Kalindjian SB, Philpott KL. Principles of early drug discovery. Br J Pharmacol 2011; 162: 1239–1249. 21091654 10.1111/j.1476-5381.2010.01127.x PMC3058157

[bib32] 32Linke K, Mace PD, Smith CA, Vaux DL, Silke J, Day CL. Structure of the MDM2/MDMX RING domain heterodimer reveals dimerization is required for their ubiquitylation in trans. Cell Death Differ 2008; 15: 841–848. 18219319 10.1038/sj.cdd.4402309

[bib33] 33Hemann MT, Bric A, Teruya-Feldstein J, Herbst A, Nilsson JA, Cordon-Cardo C et al. Evasion of the p53 tumour surveillance network by tumour-derived MYC mutants. Nature 2005; 436: 807–811. 16094360 10.1038/nature03845 PMC4599579

[bib34] 34Schmitt CA, Fridman JS, Yang M, Baranov E, Hoffman RM, Lowe SW. Dissecting p53 tumor suppressor functions *in vivo*. Cancer Cell 2002; 1: 289–298. 12086865 10.1016/s1535-6108(02)00047-8

[bib35] 35Schmitt CA, Rosenthal CT, Lowe SW. Genetic analysis of chemoresistance in primary murine lymphomas. Nat Med 2000; 6: 1029–1035. 10973324 10.1038/79542

[bib36] 36Yu J, Zhang L, Hwang PM, Kinzler KW, Vogelstein B. PUMA induces the rapid apoptosis of colorectal cancer cells. Mol Cell 2001; 7: 673–682. 11463391 10.1016/s1097-2765(01)00213-1

[bib37] 37Nakano K, Vousden KH. PUMA, a novel proapoptotic gene, is induced by p53. Mol Cell 2001; 7: 683–694. 11463392 10.1016/s1097-2765(01)00214-3

[bib38] 38Tovar C, Graves B, Packman K, Filipovic Z, Higgins B, Xia M et al. MDM2 small-molecule antagonist RG7112 activates p53 signaling and regresses human tumors in preclinical cancer models. Cancer Res 2013; 73: 2587–2597. 23400593 10.1158/0008-5472.CAN-12-2807

[bib39] 39Ding Q, Zhang Z, Liu JJ, Jiang N, Zhang J, Ross TM et al. Discovery of RG7388, a potent and selective p53-MDM2 inhibitor in clinical development. J Med Chem 2013; 56: 5979–5983. 23808545 10.1021/jm400487c

[bib40] 40Zhang Z, Chu XJ, Liu JJ, Ding Q, Zhang J, Bartkovitz D et al. Discovery of potent and orally active p53-MDM2 inhibitors RO5353 and RO2468 for potential clinical development. ACS Med Chem Lett 2014; 5: 124–127. 24900784 10.1021/ml400359z PMC4027646

[bib41] 41Wang YV, Leblanc M, Wade M, Jochemsen AG, Wahl GM. Increased radioresistance and accelerated B cell lymphomas in mice with Mdmx mutations that prevent modifications by DNA-damage-activated kinases. Cancer Cell 2009; 16: 33–43. 19573810 10.1016/j.ccr.2009.05.008 PMC2758524

[bib42] 42Garcia D, Warr MR, Martins CP, Brown Swigart L, Passegue E, Evan GI. Validation of MdmX as a therapeutic target for reactivating p53 in tumors. Genes Dev 2011; 25: 1746–1757. 21852537 10.1101/gad.16722111 PMC3165938

[bib43] 43Graves B, Thompson T, Xia M, Janson C, Lukacs C, Deo D et al. Activation of the p53 pathway by small-molecule-induced MDM2 and MDMX dimerization. Proc Natl Acad Sci USA 2012; 109: 11788–11793. 22745160 10.1073/pnas.1203789109 PMC3406834

[bib44] 44Oren M. Regulation of the p53 tumor suppressor protein. J Biol Chem 1999; 274: 36031–36034. 10593882 10.1074/jbc.274.51.36031

[bib45] 45Lev Bar-Or R, Maya R, Segel LA, Alon U, Levine AJ, Oren M. Generation of oscillations by the p53-Mdm2 feedback loop: a theoretical and experimental study. Proc Natl Acad Sci USA 2000; 97: 11250–11255. 11016968 10.1073/pnas.210171597 PMC17186

[bib46] 46Lozano G, Zambetti GP. What have animal models taught us about the p53 pathway? J Pathol 2005; 205: 206–220. 15643668 10.1002/path.1704

[bib47] 47Koduru PR, Raju K, Vadmal V, Menezes G, Shah S, Susin M et al. Correlation between mutation in P53, p53 expression, cytogenetics, histologic type, and survival in patients with B-cell non-Hodgkin's lymphoma. Blood 1997; 90: 4078–4091. 9354678

[bib48] 48Sosin AM, Burger AM, Siddiqi A, Abrams J, Mohammad RM, Al-Katib AM. HDM2 antagonist MI-219 (spiro-oxindole), but not Nutlin-3 (cis-imidazoline), regulates p53 through enhanced HDM2 autoubiquitination and degradation in human malignant B-cell lymphomas. J Hematol Oncol 2012; 5: 57. 22989009 10.1186/1756-8722-5-57 PMC3473265

[bib49] 49Li Q, Zhang Y, El-Naggar AK, Xiong S, Yang P, Jackson JG et al. Therapeutic efficacy of p53 restoration in Mdm2-overexpressing tumors. Mol Cancer Res 2014; 12: 901–911. 24598047 10.1158/1541-7786.MCR-14-0089 PMC4058386

[bib50] 50Giono LE, Manfredi JJ. Mdm2 is required for inhibition of Cdk2 activity by p21, thereby contributing to p53-dependent cell cycle arrest. Mol Cell Biol 2007; 27: 4166–4178. 17371838 10.1128/MCB.01967-06 PMC1900019

[bib51] 51Polyak K, Waldman T, He TC, Kinzler KW, Vogelstein B. Genetic determinants of p53-induced apoptosis and growth arrest. Genes Dev 1996; 10: 1945–1952. 8756351 10.1101/gad.10.15.1945

[bib52] 52Fridman JS, Hernando E, Hemann MT, de Stanchina E, Cordon-Cardo C, Lowe SW. Tumor promotion by Mdm2 splice variants unable to bind p53. Cancer Res 2003; 63: 5703–5706. 14522887

[bib53] 53Biderman L, Poyurovsky MV, Assia Y, Manley JL, Prives C. MdmX is required for p53 interaction with and full induction of the Mdm2 promoter after cellular stress. Mol Cell Biol 2012; 32: 1214–1225. 22290440 10.1128/MCB.06150-11 PMC3302446

[bib54] 54Stad R, Little NA, Xirodimas DP, Frenk R, van der Eb AJ, Lane DP et al. Mdmx stabilizes p53 and Mdm2 via two distinct mechanisms. EMBO Rep 2001; 2: 1029–1034. 11606419 10.1093/embo-reports/kve227 PMC1084126

[bib55] 55Herman AG, Hayano M, Poyurovsky MV, Shimada K, Skouta R, Prives C et al. Discovery of Mdm2-MdmX E3 ligase inhibitors using a cell-based ubiquitination assay. Cancer Discov 2011; 1: 312–325. 22586610 10.1158/2159-8290.CD-11-0104 PMC3353153

[bib56] 56Roxas-Duncan V, Enyedy I, Montgomery VA, Eccard VS, Carrington MA, Lai H et al. Identification and biochemical characterization of small-molecule inhibitors of Clostridium botulinum neurotoxin serotype A. Antimicrobial Agents Chemother 2009; 53: 3478–3486. 10.1128/AAC.00141-09 PMC2715594 19528275

[bib57] 57Moret V, Laras Y, Cresteil T, Aubert G, Ping DQ, Di C et al. Discovery of a new family of bis-8-hydroxyquinoline substituted benzylamines with pro-apoptotic activity in cancer cells: synthesis, structure-activity relationship, and action mechanism studies. Eur J Med Chem 2009; 44: 558–567. 18485536 10.1016/j.ejmech.2008.03.042

[bib58] 58Caglic D, Krutein MC, Bompiani KM, Barlow DJ, Benoni G, Pelletier JC et al. Identification of clinically viable quinolinol inhibitors of botulinum neurotoxin A light chain. J Med Chem 2014; 57: 669–676. 24387280 10.1021/jm4012164 PMC3983388

[bib59] 59Ling X, Xu C, Fan C, Zhong K, Li F, Wang X. FL118 induces p53-dependent senescence in colorectal cancer cells by promoting degradation of MdmX. Cancer Res 2014; 74: 7487–7497. 25512388 10.1158/0008-5472.CAN-14-0683 PMC4448973

[bib60] 60Wlodarska I, Aventin A, Ingles-Esteve J, Falzetti D, Criel A, Cassiman JJ et al. A new subtype of pre-B acute lymphoblastic leukemia with t(5;12)(q31q33;p12), molecularly and cytogenetically distinct from t(5;12) in chronic myelomonocytic leukemia. Blood 1997; 89: 1716–1722. 9057655

